# Diagnostic and prognostic value of microRNA-2355-3p and contribution to the progression in lung adenocarcinoma

**DOI:** 10.1080/21655979.2021.1952367

**Published:** 2021-08-01

**Authors:** Yanan Zhao, Wenlong Zhang, Yang Yang, Enyong Dai, Yuansong Bai

**Affiliations:** Department of Oncology and Hematology, China-Japan Union Hospital of Jilin University, Changchun, Jilin, China

**Keywords:** miR-2355-3p, *ZCCHC14*, clinical significance, biological function, lung adenocarcinoma

## Abstract

The aim of this study was to delve into the clinical significance and biological function of miR-2355-3p in LUAD. Tissues and blood samples from 116 LUAD patients and blood samples of 90 healthy volunteers were collected. The relative expression of miR-2355-3p was evaluated by quantitative real-time polymerase chain reaction (qRT-PCR). The receiver operating curve (ROC) was plotted for diagnostic value estimation. Kaplan–Meier survival curves were constructed, and multivariate survival analyses were performed for prognostic value estimation. A luciferase reporter assay was carried out to confirm the interaction of miR-2355-3p and *ZCCHC14*. The CCK-8 and transwell assays were conducted to explore the function of miR-2355-3p on LUAD cells. Rescue experiments were performed to verify the interaction. miR-2355-3p showed an upregulated expression in the samples of LUAD patients. For diagnostic value estimation, the AUC was 0.905 with a sensitivity was 84.5% and specificity of 83.3%. For the estimation of prognostic value, the *P*-value of log-rank test on K-M curves was 0.002 and 0.006 for overall survival and progression survival, respectively. Based on multivariate Cox regression analysis, miR-2355-3p was a powerful prognostic tool with a *P*-value of 0.027. *ZCCHC14* has binding sites with miR-2355-3p, an expression level, and luciferase activity negatively correlated with miR-2355-3p expression. Knockdown of miR-2355-3p inhibited proliferation, migration, and invasion of LUAD cells, but *ZCCHC14* can rescue this inhibition. miR-2355-3p has the potential to be a diagnostic marker and prognostic marker for LUAD. Inhibition of miR-2355-3p in LUAD cells can suppress the progression of LUAD at least partly by direct targeting *ZCCHC14*.

## Graphical abstract

**Figure:**
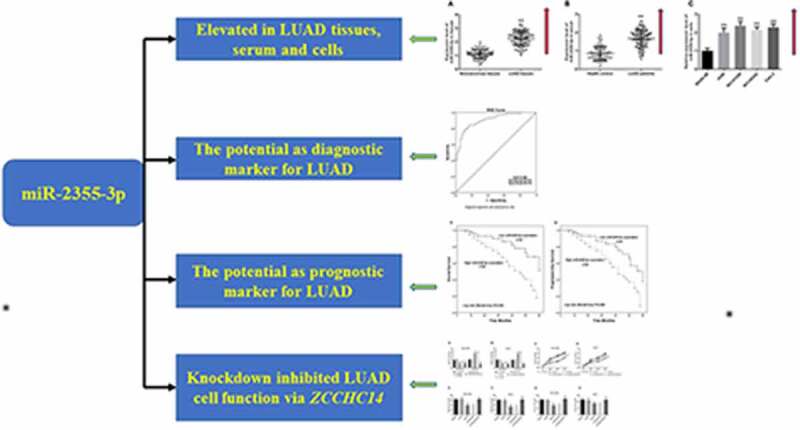

## Introduction

Lung cancer ranked first in incidence and mortality rates among men globally, third in incidence, and second in causes of deaths for women in 2020 according to a database generated by the International Agency for Research on Cancer (IARC). Lung adenocarcinoma (LUAD) accounts for approximately 60% of non-small cell lung cancer (NSCLC) which is the most common histological subtype of lung cancer [1]. In China, lung cancer is still the first cause of cancer-related death due to its highest incidence of cancer [2]. Five-year stage-specific survival rates of lung cancer patient survival ranged largely from 73% to 13% depending upon the clinical and pathologic stages at diagnosis [3,4]. To reduce the worrying rise in deaths and the high prevalence of lung cancer, various early-detection methods and prognosis prediction methods have been introduced to control mortality [3,5]. Chest radiography and low-dose CT screening currently approach for the early diagnosis of lung cancer. However, chest radiography lacks high sensitivity, while low-dose CT screening usually presents a false-positive rate up to 96.4% because confusing with non-tumorous solitary pulmonary nodules due to its high sensitivity [6,7]. Thus, noninvasive diagnostic methods that successfully balance sensitivity and specificity seem extremely important. Moreover, more reliable and easily measured predictors of a prognosis needed to be developed further.

Benefiting from rapid genetic sequencing, an increasing amount of tumor-specific molecular biomarkers such as microRNAs (miRNAs/miRs) can refine diagnostic methods and prognostic estimation beyond traditional ones [8,9]. Though short and non-coding, miRNAs can bind to target mRNA at 3ʹ-untranslated region (3ʹ-UTR), 5ʹ-UTR, or ORF sites, leading to transcriptional repression or translational inhibition [10]. MicroRNA expression profiles differ disease samples from normal tissues and thus provide the basis as diagnostics and prognosis markers [11,12]. For instance, miR-125b-5p shows differential expression in LUAD tissues enough to distinguish LUAD from adjacent tissues and can be a prognostic and diagnostic biomarker for LUAD [13]. Serum miR-1290 was significantly upregulated in LUAD patients compared to healthy controls and was identified to be a potential diagnostic and prognostic biomarker for LUAD [14]. Given the fact that miR-2355-3p was a differentially expressed miRNA in LUAD [15], it is necessary to investigate the role of miR-2355-3p in LUAD.

Thus, to clarify the detailed role of miR-2355-3p in LUAD, we first determined the expression level of miR-2355-3p in LUAD. Then, the value of miR-2355-3p as a biomarker for LUAD was delved into in the present study. The diagnostic value was estimated based on the expression level in LUAD and healthy serum. The prognostic value was accessed by the survival time in the high and low miR-2355-3p expression groups. The target gene of miR-2355-3p was found might be *ZCCHC14* and would be verified. The effect of miR-2355-3p on LUAD cells was investigated.

## Methods and materials

### Subjects

The experimental protocols of this study were approved by the Ethics Committee of China-Japan Union Hospital of Jilin University. Each participant signed written informed consent. From patients with LUAD who underwent lung surgery at China-Japan Union Hospital of Jilin University between January 2011 and December 2015, 116 pairs of frozen LUAD tissues and corresponding non-cancerous tissues were collected, along with their serum samples. No patients had been administered preoperative radiation, chemotherapy, or any other therapy related to lung cancer. TNM staging was unified based on the seventh edition of the International Association for the Study of Lung Cancer system for NSCLC before being subjected to subsequent analysis [16]. The follow-up duration for LUAD patients was 60 months after the surgery, and their survival information was obtained from the medical records for the subsequent survival analysis.

Meanwhile, 90 cases of blood serum were obtained from healthy volunteers in agreement with the knowledge. Blood (20 ml) was collected from all 116 patients and 90 healthy controls and incubated at room temperature for 1.5 h. Then, the serum was obtained after centrifugation at 1,400 × g and then stored at −80°C before use.

### Cell lines and transfection

The utilized human LUAD cell lines A549 (ATCC® CCL-185™), NCI-H1299 (ATCC® CRL-5803™), NCI-H2342 (ATCC® CRL-5941™), and Calu-3 (ATCC® HTB-55™), and an immortalized human bronchial epithelial cell line (BEAS-2B) were obtained from the American Type Culture Collection (ATCC) and grown at 37°C in a humidified incubator filled with 5% CO_2_. The LUAD cell lines were grown in RPMI 1640 medium (GIBCO, Grand Island, NY) supplemented with 10% qualified fetal bovine serum (Gibco by Life Technologies, Australia).

miR-2355-3p inhibitor (anti-miR, 5ʹ-AUCUCCAAACAGCAAGGACAAU-3ʹ), miR-2355-3p inhibitor negative control (anti-NC, 5ʹ-CCAUCAGUCCCAAAUCCA-3ʹ), predesigned short-interfering RNA (siRNA) targeting *ZCCHC14* (si-*ZCCHC14*), and control siRNA (si-NC) were purchased from Sino Biological Inc. (Beijing, China). Cells were transfected with the above constructs individually or combinedly, just as anti-miR and si-*ZCCHC14*, or anti-miR and si-NC according to Lipofectamine 3000 (Invitrogen, USA) transfection manual. Transfected cells were harvested 24 h later for proliferation assay, migration, and invasion assay, and 72 h later to examine RNA expression.

### Assessment of miRNA and mRNA expression by qRT-PCR

TRIzol® reagent (Invitrogen; Thermo Fisher Scientific Inc., USA) was utilized to extract total RNA from LUAD tissues (Ground in liquid nitrogen beforehand), adjacent non-cancerous tissues, serum, and *in vitro* cultured cells [17]. For miR-2355-3p expression assay, a specific TaqMan microRNA assay primer with an Applied Biosystems® TaqMan® MicroRNA Reverse Transcription Kit (Life Technologies) was used to transcribe, and a high-capacity RNA-to-cDNA kit (Life Technologies) was used for mRNA analysis. qRT-PCR was performed using a QuantStudio 3 (Applied Biosystems). GAPDH was used as the internal control for *ZCCHC14* mRNA, and U6 was used for miR-2355-3p. Data were analyzed by the 2^−ΔΔCt^ method.

### CCK-8 kit for Cell proliferation assay

Transfected NCI-H1299 and Calu-3 cells were collected and prepared in a cell suspension. Then, the cell suspension was inoculated into a 96-well plate with the volume of 100 μl per well (2 × 10^3^ cells/well) and then pre-incubated the plate in a humidified incubator filled with 5% CO_2_ at 37°C [18,19]. At 0, 24, 48, 72 h, 10 μl of the CCK-8 solution was added to each well of the plate carefully in case of introducing bubbles. The cells with CCK-8 solution were incubated for an extra 2 hours in the incubator. When incubation finished, the absorbance at 450 nm was measured on the Thermo Scientific Varioskan LUX Multimode Reader (Thermo Fisher Scientific, Vantaa, Finland).

### Transwell migration and invasion assay

Transwell Permeable Supports (8.0 μm, Corning, USA) were utilized for cell migration and invasion assay [19]. For migration assay, cells were harvested 24 h after transfection. 5 × 10^4^ of NCI-H1299 or 2 × 10^5^ of Calu-3 serum-starved cells suspended in 100 μl serum-free medium were seeded on top of the filters, and a medium containing 10% FBS was added to the lower chamber. For invasion assay, the Transwell inserts were pre-coated with Matrigel Matrix (BD Biosciences, USA), and others were the same as migration assay. After incubation for 24 h, cells that did not pass through the membrane were removed, and the migratory LUAD cells on the bottom surface of the membrane can be fixed with 4% paraformaldehyde, stained with a 0.4% crystal violet solution. The migratory or invading cells were imaged with a Leica DC 300 F microscopy (Leica, Germany), and counted in five random fields.

### Dual-luciferase reporter assay

The 3ʹ-UTR sequence of *ZCCHC14* (WT-*ZCCHC14*) and its mutant sequence of miR-2355-3p binding sites (MUT- *ZCCHC14*) were cloned into pmirGLO Dual-luciferase miRNA Target Expression Vectors (GenePharama, Shanghai, China). Cells (NCI-H1299) were seeded in 96-well plates to settle overnight, and then co-transfected with WT-*ZCCHC14* (or MUT-*ZCCHC14*) reporter plasmid and anti-2355-3p or anti-NC, respectively. The luciferase activities were performed with the Dual-Lucifer Reporter Assay System (Promega, USA) and normalized to renilla luciferase activity, respectively [20]. Experiments were performed in triplicate.

### Statistical analysis

The data were given as mean ± standard deviation (SD). Two-tailed Student’s t-test or one-way ANOVA was introduced to access the difference among two or more groups. For diagnostic value estimation, receiver operating characteristic (ROC) curves were used to evaluate the specificity and sensitivity of miR-2355-3p for LUAD diagnosis. For survival analysis, Kaplan‐Meier survival curves were constructed, and differences among groups were tested with the log-rank test. Multivariate survival analyses were performed with the likelihood test. Differences were considered to be significant if *P* < 0.05. Statistical analyses were performed in IBM SPSS 23 (SPSS Inc., Chicago, IL) and GraphPad Prism 7(GraphPad Software Inc., San Diego, USA).

## Results

In the forthcoming study, we focus on the investigation of the diagnostic and prognostic significance of miR-2355-3p and the potential molecular mechanism of it in LUAD, in the hope of discovery of new biomarker and therapeutic targets for LUAD. We detected miR-2355-3p level was ascendant in LUAD blood, tissues, and cells using qRT-PCR and verified that this increase was an effective diagnostic and prognostic tool for LUAD. Furtherly, the CCK-8 and Transwell assays were utilized to explore the role of miR-2355-3p in LUAD cellular function. Using prediction tools and correlation analysis, it is reasonable to infer that miR-2355-3p might be involved in the progression of LUAD through moderating *ZCCHC14*.

### Expression of miR-2355-3p in LUAD tissues, serums, and cells

**Figure 1.**
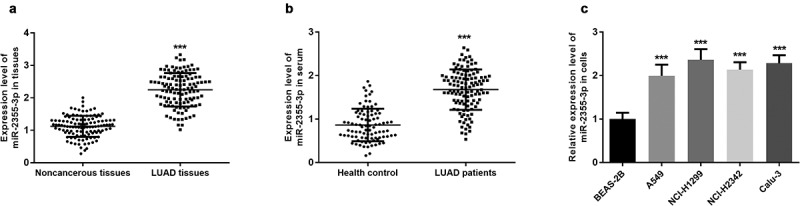
The expression level of miR-2355-3p in LUAD tissues, serum, and cells measured by qRT-PCR. (a). miR-2355-3p expression was relatively higher in LUAD tissues compared with non-cancerous tissues. (***P < 0.001). (b). miR-2355-3p expression was relatively higher in LUAD patient serums compared with healthy controls. (***P < 0.001). c) miR-2355-3p expression was relatively higher in CC cells compared with normal cells. (***P < 0.001)

qRT-PCR was performed to determine the relative expression of miR-2355-3p in cancerous or normal tissues, serums, and cells. Compared with non-cancerous tissues, miR-2355-3p was upregulated in LUAD tissues (*P* < 0.001, Figure 1(a)). A significantly higher expression of miR-2355-3p was found in the serum samples obtained from LUAD patients than those from healthy individuals (*P* < 0.001, Figure 1(b)). Similarly, the expressions of miR-2355-3p in four LUAD cell lines were significantly higher than the normal lung cell line (all *P* < 0.001, Figure 1(c)). Among the four kinds of LUAD cells, elevated expression of miR-2355-3p was more prominent in NCI-H1299 and Calu-3 cells.

### Correlation of miR-2355-3p with subject characteristics

The collected tissue samples were divided into a low group (n = 54) and a high group (n = 62) based on the mean cutoff value of the miR-2355-3p expression in LUAD tissues. The serum samples were divided into a low group (n = 56) and a high group (n = 60) according to the mean cutoff value of the miR-2355-3p expression in LUAD serum. Based on the analysis of the chi-square test, the relationship between miR-2355-3p expression in LUAD tissues or serums and the clinical-pathological factors of LUAD patients was investigated. Based on the results shown in Table 1, the expression of miR-2355-3p in LUAD tissues is affected by the N stage (*P* = 0.018) and M stage (*P* = 0.026). In serum, miR-2355-3p was associated with N stage (*P* = 0.024, Table 2).

**Table 1.** Correlation of the miR-2355-3p expression in tumor tissues with clinical characteristics of LUAD patients. High miR-2355-3p in LUAD tissues was associated with high N stage and M stage (*P* < 0.05)

Parameters	Cases (n = 116)	miR-2355-3p expression		*P*
		Low (n = 54)	High (n = 62)	
Age				0.690
≤ 55	56	25	31	
> 55	60	29	31	
Gender				0.748
Female	62	28	34	
Male	54	26	28	
Smoking status				
Nonsmoker	55	27	28	0.603
Smoker	61	27	34	
Tumor size				0.055
≤ 3 cm	62	34	28	
> 3 cm	54	20	34	
Pathological stage				0.170
I	63	33	30	
II–IV	53	21	32	
N stage				0.018*
I	75	41	34	
II–IV	41	13	28	
M stage				0.026*
I	107	53	54	
II–IV	9	1	8	

*P < 0.05.

**Table 2.** Correlation of the miR-2355-3p expression in serum with clinical characteristics of LUAD patients. High miR-2355-3p in LUAD tissues was associated with high N stage (*P* < 0.05)

Parameters	Cases (n = 116)	miR-2355-3p expression		*P*
		Low (n = 56)	High (n = 60)	
Age				0.465
≤ 55	56	29	27	
> 55	60	27	33	
Gender				0.472
Female	62	28	34	
Male	54	28	26	
Smoking status				
Nonsmoker	55	29	26	0.362
Smoker	61	27	34	
Tumor size				0.130
≤ 3 cm	62	34	28	
> 3 cm	54	22	32	
Pathological stage				0.087
I	63	35	28	
II–IV	53	21	32	
N stage				0.024*
I	75	42	33	
II–IV	41	14	27	
M stage				0.103
I	107	54	53	
II–IV	9	2	7	

*P < 0.05.

### Diagnostic value of miR-2355-3p for LUAD

**Figure 2.**
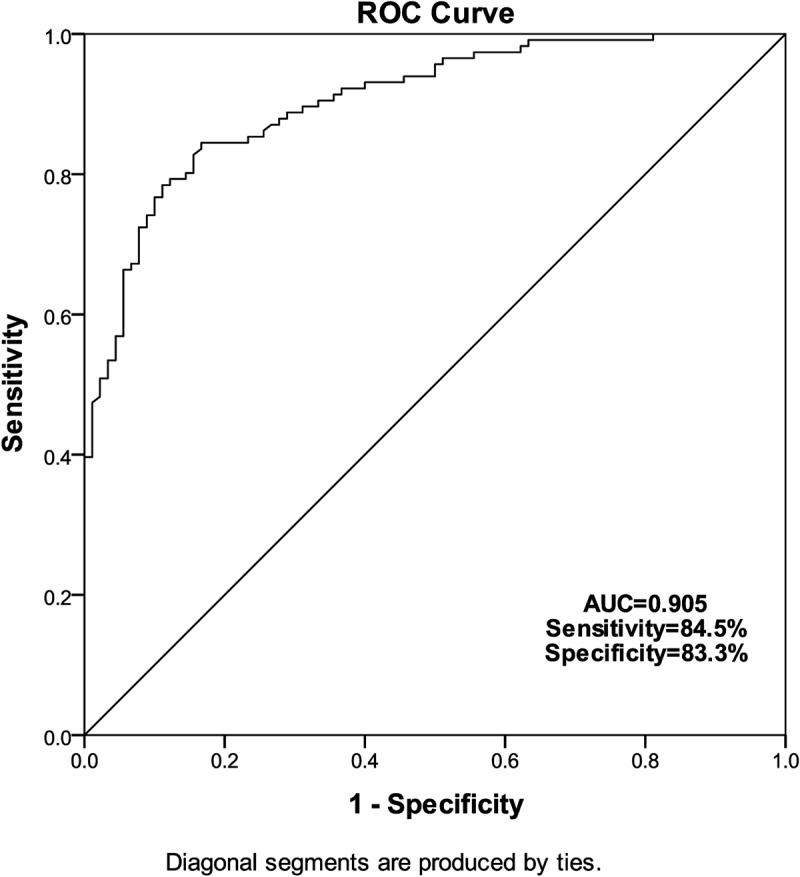
The ROC curve was used to analyze the diagnostic value of miR-2355-3p in LUAD. (a) The AUC is 0.905, sensitivity is 84.5%, specificity is 83.3%

Furthermore, ROC curve analyses were performed to estimate the predictive power of serum miR-2355-3p in LUAD diagnosis. As illustrated in Figure 2, miR-2355-3p is efficient in distinguishing LUAD patients from healthy controls. It was observed that miR-2355-3p had an AUC of 0.905 (sensitivity = 84.5%, specificity = 83.3%). These results implied that miR-2355-3p may be a promising diagnostic biomarker for LUAD.

### Prognostic value of miR-2355-3p for LUAD

**Figure 3.**
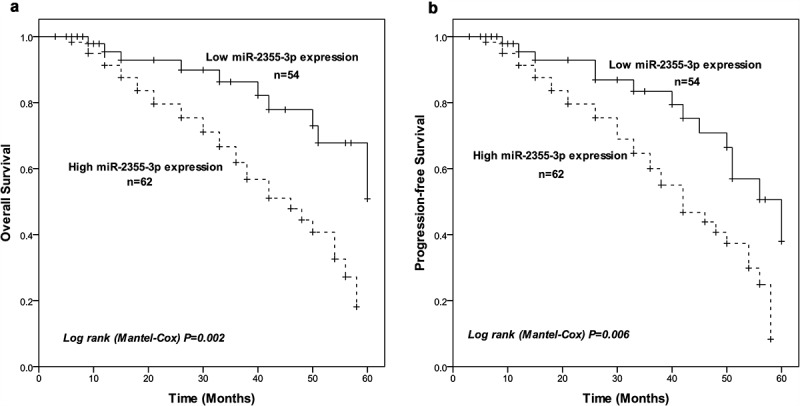
Kaplan-Meier survival analysis. (a) Overall survival curves constructed with the expression of miR-2355-3p. Log-rank test *P* = 0.002. (b) Progression-free survival curves constructed with the expression of miR-2355-3p. Log-rank test *P* = 0.006

Over a 5-year follow-up period, the Kaplan–Meier curve was drawn to present the relationship of miR-2355-3p expression and prognosis in LUAD patients. The prognosis for the higher miR-2355-3p expression group seems to be worse than for the lower expression group in overall survival outcome (log-rank test, *P* = 0.002, Figure 3(a)). Likewise, patients with high miR-2355-3p expression showed shorter progression-free survival (log-rank test, *P* = 0.006, Figure 3(b)). Further, multivariate Cox regression analysis was introduced to identify statistically significant prognostic factors, and the result revealed up-regulation of miR-2355-3p (HR = 2.465, 95%CI: 1.109 − 5.481, *P* = 0.027) can be a significant independent prognostic factor for LUAD patients (Table 3).

**Table 3.** Multivariate Cox analysis of clinical characteristics correlated to overall survival. miR-3677-3p in LUAD tissues was found to be a powerful predictor of prognosis of overall survival

Characteristics	Multivariate analysis		
	hazard ratio	95% Confidence interval	*P*
miR-3677-3p in LUAD tissues (High vs. Low)	2.465	1.109–5.481	0.027
Age (> 55 vs. ≤ 55)	1.459	0.762–2.795	0.255
Gender (Male vs. Female)	1.728	0.888–3.364	0.274
Smoking status (Smoker vs. Nonsmoker)	1.105	0.567–2.153	0.368
Tumor size (> 3 cm vs. ≤ 3 cm)	1.367	0.693–2.699	0.107
Pathological stage (II–IV vs. I)	1.860	0.965–3.586	0.768
N stage (II–IV vs. I)	1.867	0.961–3.852	0.064
M stage (II–IV vs. I)	2.034	1.075–4.954	0.029

### ZCCHC14 was a target gene of miR-2355-3p

**Figure 4.**
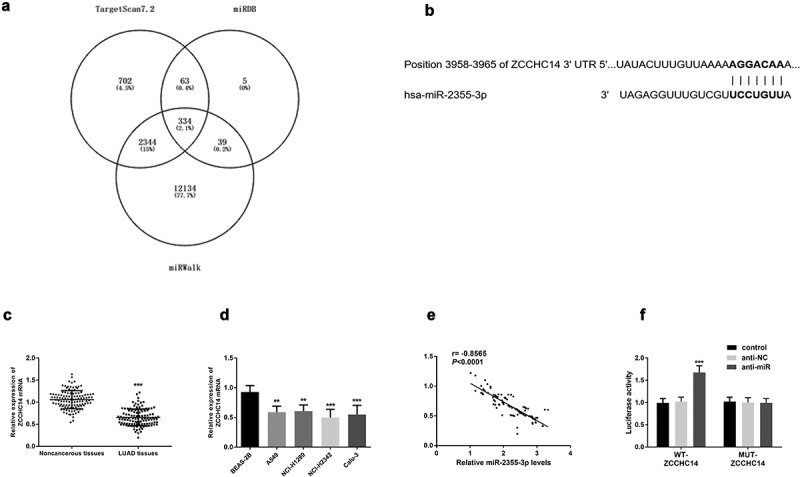
*ZCCHC14* is a target gene of miR-2355-3p. (a) Predicted binding sites of miR-2355-3p and *ZCCHC14*. (b) Expression of *ZCCHC14* was downregulated in LUAD tissues. (***P < 0.001). (c) Expression of *ZCCHC14* was downregulated in LUAD cells. (**P < 0.01, ***P < 0.001). (d) The correlation between the expression of miR-2355-3p and *ZCCHC14* is negative in LUAD tissues. (r = −0.8565, *p* < 0.0001). (e) Dual-luciferase reporter assay was performed in NCI-H1299. (***P < 0.001)

According to integration analysis from TargetScan (http://www.targetscan.org/vert_72/), miRWalk (http://mirwalk.umm.uni-heidelberg.de/) and miRDB (http://mirdb.org/) database (Figure 4(a)), *ZCCHC14* was one gene of miR-2355-3p with seven consecutive binding sites in the 3ʹUTR of *ZCCHC14* (Figure 4(b)). Moreover, *ZCCHC14* was significantly downregulated in LUAD tissue and cells compared with normal ones (*P* < 0.001, Figure 4(b, c)). Furthermore, the expression of *ZCCHC14* is negatively correlated with that of miR-2355-3p in LUAD tissues (r = −0.8565, *P* < 0.001, Figure 4(d)). To verify that directly, luciferase activity was evaluated after co-transfection of miR-2355-3p and *ZCCHC14* 3ʹ UTR wild type or mutated luciferase plasmids. As shown in Figure 4(e), co-transfection with WT-*ZCCHC14* vector and miR-2355-3p inhibitor can increase luciferase reporter activity significantly compared with the control group (*P* < 0.001).

### miR-2355-3p promotes LUAD cell proliferation, migration, and invasion, which can be reversed by ZCCHC14

**Figure 5.**
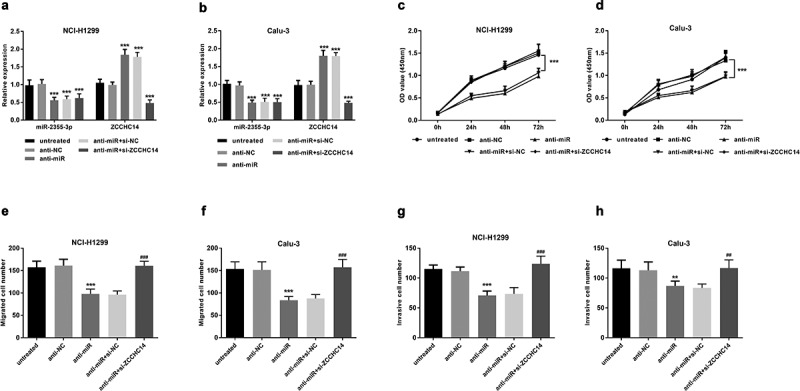
Downregulation of miR-2355-3p can inhibit the proliferative, migrative, and invasive capacity of LUAD cells, but *ZCCHC14* can reverse this effect. (a) and (b) The results of miR-2355-3p and *ZCCHC14* expression showed that the transfection was successful. (***P < 0.001). (c) and (d) Downregulated miR-2355-3p inhibits NCI-H1299 and Calu-3 cell proliferation, but si-*ZCCHC14* abolished the suppressive effect. (***P < 0.001). (e) and (f) NCI-H1299 and Calu-3 cell migration were inhibited by knocking down miR-2355-3p expression but rescued by si-*ZCCHC14*. (***P < 0.001 ratio to anti-NC; ^###^*P* < 0.001 ratio to anti-miR+si-NC). (g) and (h) LUAD cell invasion was repressed by knocking down miR-2355-3p expression but recuperated by *ZCCHC14* inhibitor. (**P < 0.01, ***P < 0.001 ratio to anti-NC; ^##^*P* < 0.01, ^###^*P* < 0.001 ratio to anti-miR+si-NC)

To illustrate whether miR-2355-3p exerts biological functions on LUAD cells, a specific inhibitor against miR-2355-3p was designed and transfected into NCI-H1299 and Calu-3 cell lines. miR-2355-3p levels decreased after transfection with anti-miR and *ZCCHC14* mRNA levels decreased after transfection with siRNA (*P* < 0.001, Figure 5(a, b)). As determined via CCK-8 assay, inhibition of miR-2355-3p expression resulted in a significant suppression in NCI-H1299 and Calu-3 cell proliferation, while *ZCCHC14* siRNA can reverse this result (*P* < 0.001, Figure 5(c, d)). To further explore whether miR-2355-3p works as a promotor rather than an inhibitor in LUAD cell growth, a transwell assay was conducted to detect cell migration and invasion ability. As shown in Figure 5(e–h), the cellular migration and invasion were reduced by the miR-2355-3p inhibitor, but the restriction was relieved when *ZCCHC14* siRNA exists at the same time. So, miR-2355-3p can promote LUAD cell proliferative, migratory, and invasive capacity, which can be reversed by *ZCCHC14*.

## Discussion

In the previous study, many miRNAs have been identified as differentially expressed in cancerous and normal tissues [21]. For instance, 5 microRNAs were significantly upregulated, and 12 microRNAs were downregulated in oral squamous cell carcinoma [22]. The miRNA profiles of 24 lung adenocarcinoma and paired non-tumor tissues in Xuanwei, China, revealed 4 upregulated miRNAs and 4 downregulated miRNAs, which might be used as biomarkers for diagnosis and/or prognosis for lung cancer [23]. miR-2355 has been reported to be upregulated in coronary artery disease, nonresponder samples of head and neck squamous cell carcinoma, and cervical cancer [24,25]. In the current study, a significant increase of miR-2355-3p expression was observed in the tissues, serums, and cells in patients with LUAD and non-cancerous ones. This differential expression provides a basis for the feasibility of miR-2355-3p as a diagnostic or prognostic marker for LUAD.

Due to the unsatisfactory sensitivity and specificity of the traditional screen strategy of lung cancer, molecular biomarkers have gradually become an adjunctive tool for lung cancer screening [26,27]. Among them, miRNAs stable in body fluids, such as blood, have emerged as fascinating potential biomarkers for cancer diagnosis, which was enhanced by the feasibility and less invasion in detecting [28]. Currently, numerous miRNAs have been discovered as a diagnostic tool for LUAD. For example, 13 DEmiRNAs were identified as optimal LUAD-specific biomarkers with a great diagnostic value [29]. Expressions of serum ex-miR-21-5p, −126-3p, and −140-5p were up-regulated in LUAD patients and have a great potential to serve as highly sensitive and repeatable biomarkers for the early diagnosis of LUAD [30]. The differential expression of miR-2355-3p inspired us to explore its diagnostic value in serum for LUAD. With the use of ROC curves, the significance of miR-2355-3p as a diagnosis factor was confirmed with reliable sensitivity and specificity. The results were generally consistent with the profile of the abnormally expressed miRNAs including miR-2355-3p in NSCLC. In summary, our study identified miR-2355-3p may serve as potential diagnostic biomarkers for LUAD.

Differentially expressed miRNAs as oncogenes and tumor suppressor genes play major roles in lung cancer prognosis. Low expression of let-7 microRNA by a meta-analysis was revealed as a poor prognosis predictive factor in lung cancer patients [31]. High miR-130b expression was significantly associated with the unfavorable prognosis of LUAD and may serve as a potential therapeutic strategy for lung cancer [32]. So further, the prognostic significance of miR-2355-3p in LUAD tissues was estimated by Kaplan–Meier survival curves and multivariate survival analyses. The results implied overexpression miR-2355-3p expression in LUAD tissues was significantly associated with worse clinicopathological parameters of LUAD. Moreover, LUAD patients in the high tissue miR-2355-3p expression group had significantly shorter overall survival and progression-free survival than those in the low miR-2355-3p expression group. miR-2355-3p expression in surgical LUAD tissues was demonstrated to be an independent prognostic factor for LUAD. Consistent with our results, a CWx framework developed to predict prognostic miRNAs data from 192 LUAD patients obtained from TCGA, miR-2355-3p was identified to be associated with the survival of LUAD patients [33]. Taken together, miR-2355-3p might be a promising biomarker for prognosis prediction of LUAD.

Depending on the type of cancer, miR-2355 has been shown to act as both a tumor suppressor and an oncogene. In a breast cancer cell, the expression level of miR-2355 was significantly decreased when cells were treated with rh-endostatin/bevacizumab [34]. miR-2355 was validated to be able to bind with DDX11-AS1 and inhibit cell proliferation in bladder cancer [35]. In this study, we examined miR-2355-3p expression in tissues and cells and found miR-2355-3p is increased in LUAD and associated with progression-free survival, whereas this increased expression can promote proliferation, migration, and invasion of LUAD cells *in vitro*. miRNAs may act as either an oncogene or cancer suppressor in different cancers, due to their dysregulation trends. MiRNA-485-5p was found by Gao et al. to be decreased in SCLC tissues compared to normal ones and inhibited the growth and metastasis of SCLC cells by targeting FLOT2, which may be as a tumor suppressor [36]. However, this study has not made a thorough inquiry into the clinical significance of miR-485-5p, such as diagnostic value or prognostic value. In addition, low expression of miRNA, if the expression level is too low, is not easy to determine. In our study, miR-2355-3p was upregulated in LUAD, which is convenient for detection, and the clinical significance of miR-2355-3p in LUAD was accessed thoroughly. Given that fundamental characteristics of cancer are sustained in cell proliferation and activated in metastasis, miR-2355-3p may be an oncogene in LUAD.

Because miRNAs function as gene regulators, identifying miRNA targets can provide more information on the role of miR-2355-3p in LUAD. By bioinformatic analysis, *ZCCHC14* was selected as the potential target gene of miR-2355-3p, which was verified by binding sites, luciferase activity change, and the correlation of expressions. Moreover, *ZCCHC14* can reverse the influence of miR-2355-3p on LUAD cellular function. So, miR-2355-3p may exert important functions in LUAD cellular biological processes via directly suppressing the expression of *ZCCHC14*.

## Conclusions

In conclusion, miR-2355-3p showed an upregulated expression in LUAD, and this overexpression can distinguish the LUAD serum from normal serum. Moreover, LUAD patients with higher miR-2355-3p levels result in an unfavorable prognosis. Inhibition of miR-2355-3p can suppress the progression of LUAD by targeting *ZCCHC14*. miR-2355-3p can be a promising noninvasive diagnostic factor and a reliable prognostic factor for LUAD, as well as a potential therapeutic target.

## Highlights

miR-2355-3p showed an elevated expression level in LUAD.

Knockdown of miR-2355-3p inhibited growth and metastasis of LUAD cells via *ZCCHC14.*

miR-2355-3p has the potential to be a diagnostic marker for LUAD.

miR-2355-3p has the potential to be a prognostic marker for LUAD.

## Data Availability

Data might be provided by the corresponding author.
